# Comparison of the Effect of Endurance, Strength, and Endurance-Strength Training on Inflammatory Markers and Adipokines Levels in Overweight and Obese Adults: Systematic Review and Meta-Analysis of Randomised Trials

**DOI:** 10.3390/healthcare10061098

**Published:** 2022-06-13

**Authors:** Aleksandra Makarewicz, Małgorzata Jamka, Jakub Geltz, Angelika Śmidowicz, Marta Kokot, Nina Kaczmarek, Edyta Mądry, Jarosław Walkowiak

**Affiliations:** 1 Department of Pediatric Gastroenterology and Metabolic Diseases, Poznan University of Medical Sciences, Szpitalna Str. 27/33, 60-572 Poznań, Poland; a.u.makarewicz@gmail.com (A.M.); mjamka@ump.edu.pl (M.J.); kuba.geltz@gmail.com (J.G.); angelika.smidowicz@gmail.com (A.Ś.); marta.kokot21@gmail.com (M.K.); nina.kaczmarek93@gmail.com (N.K.); 2 Department of Physiology, Poznan University of Medical Sciences, Święcickiego Str. 6, 60-781 Poznań, Poland; emadry@ump.edu.pl

**Keywords:** physical activity, exercises, inflammation, C-reactive protein, pro-inflammatory cytokines

## Abstract

The aim of this meta-analysis was to compare the effects of endurance, strength, and combined training on inflammatory markers and adipokine concentrations in overweight and obese adults. We performed a literature search of the Cochrane Library, PubMed, Scopus, and Web of Science databases and identified 24 randomised control trials published prior to June 2021. Our findings indicate that endurance training was significantly more beneficial than strength training in reducing C-reactive protein (CRP) (standard mean difference (SMD): −1.317, 95% confidence intervals (CI): −2.565, −0.070, *p* = 0.0385), interleukin 6 (IL-6) (SMD: −0.363, 95% CI: −0.648, −0.078, *p* = 0.0126), and visfatin (SMD: −0.618, 95% CI: −1.015, −0.222, *p* = 0.0023) concentrations. Moreover, combined training was more beneficial than strength training alone in lowering tumour necrosis factor-alpha (TNF-α) levels (SMD: 0.890, 95% CI: −0.301, 1.478, *p* = 0.0030). There were no differences between the effects of different types of training programmes on adiponectin and leptin concentrations. In conclusion, compared with strength training, endurance training is more effective in lowering CRP, IL-6, and visfatin concentrations, while combined training is more beneficial in reducing TNF-α levels in overweight and obese adults. Further studies are needed to determine which type of training has a better effect on adiponectin and leptin concentrations in this population.

## 1. Introduction

The World Health Organisation (WHO) defines being overweight or obese as having abnormal or excessive fat accumulation that presents a health risk, and, since 1997, has classified these as global epidemics [1]. These conditions are the major public health problems of modern times. Recent data show that almost 2 billion adults worldwide are overweight, of which more than 670 million are obese [2]. Obesity is associated with a high risk of morbidity and mortality, as well as reduced life expectancy [3]. Being overweight or obese increases the risk of developing multiple diseases, such as type 2 diabetes mellitus (DM2), cardiovascular disease, several types of cancers, non-alcoholic fatty liver disease, an array of musculoskeletal disorders, and poor mental health. Obesity is a chronic metabolic disease characterised by energy intake exceeding energy expenditure [1,3,4].

A large number of studies have confirmed that exercise intervention is one of the effective means to prevent and treat obesity and reduce the risk of developing concomitant diseases [3,5,6]. Physical training is known to be associated with body weight and fat mass loss and balanced body composition [5]. It has been shown that endurance training (ET), in particular, leads to numerous health benefits. ET can decrease weight and fat mass and improve glucose tolerance, concentrations of high-density lipoproteins, and endothelial functions. Therefore, ET is the most recommended type of exercise in the treatment of obesity [4,6]. However, results of recent research indicate that strength training (ST) may also be beneficial in the therapy of obesity [6]. ST promotes greater muscle mass accretion than ET, and may thus contribute to fat mass loss via an increase in the resting metabolic rate [7]. On the other hand, ET also has a positive effect on the maintenance of muscle mass and strength during body weight reduction. 

Weight gain with the accompanying increase in the amount of adipose tissue may lead to the overproduction of pro-inflammatory cytokines and adipokines, such as tumour necrosis factor-alpha (TNF-α), interleukin-6 (IL-6), visfatin, and leptin [8]. Excess adipose tissue and obesity are also associated with an increase in inflammatory markers, such as C-reactive protein (CRP) [9], and a decrease in adipokines with anti-inflammatory properties such as adiponectin [10]. Recent studies have suggested that many pro-inflammatory markers may be involved in the pathogenesis of the processes that lead to the development of hypertension, DM2, and atherosclerosis, while weight loss decreases the concentrations of those parameters [11,12]. Several studies have compared the effect of ET, ST, and combined training (CT) in overweight and obese adults [6,13]. However, these studies show inconsistent results on the effectiveness of the different types of training on inflammatory parameters and adipokine levels. Most of the studies have shown that all three types of training not only decrease the levels of CRP [14,15], pro-inflammatory cytokines (TNF-α, IL-6) [16,17], and adipokines (leptin, visfatin) [18,19], but also increase the concentrations of adiponectin [20]. On the other hand, other studies have shown the opposite effect [21,22]. Moreover, some studies have observed an increase in IL-6 [23,24] and visfatin [25] levels after training programmes. In addition, the recent meta-analysis by Zheng et al. [26] found that ET can effect a reduction in CRP, IL-6, and TNF-α in middle-aged and elderly people compared to a control group. However, there is a lack of meta-analysis comparing the effects of ET, ST, and combination training on these parameters.

Based on this consideration, we performed a systematic review and meta-analysis of randomised trials to compare the effect of endurance, strength, and combined training on inflammatory markers and adipokine levels in overweight and obese adults.

## 2. Materials and Methods

### 2.1. Protocol and Registration

This systematic review and meta-analysis was carried out and reported in accordance with the Preferred Reporting Items for Systematic Reviews and Meta-Analyses (PRISMA) [27] and the Cochrane guidelines [28]. The protocol of the study was registered in the international prospective register of systematic reviews (PROSPERO) database with the registration number CRD42020183252 [29]. No deviation from the study protocol was observed.

### 2.2. Information Sources and Search Strategy

The Cochrane Library (1908—June 2021), PubMed (1966—June 2021), Scopus (1960—June 2021), and Web of Science (1864—June 2021) databases were searched, restricted to English language articles and studies performed in humans, using MeSH terms and keywords. The search strategies used in each database are presented below.

Cochrane:

#1—(obesity OR overweight [Title, Abstract, Keyword])
#2—(endurance training OR strength training OR exercise [Title, Abstract, Keyword])
#3—#1 AND #2
#4—#3 AND (Trials AND English [Filter])

PubMed:

#1—(obesity OR overweight [MeSH Terms])
#2—(endurance training OR strength training OR exercise [MeSH Terms])
#3—#1 AND #2
#4—#3 AND (humans AND English [Filter])

Scopus:

#1—(obesity OR overweight [Article title, Abstract, Keywords])
#2—(endurance training OR strength training OR exercise [Article title, Abstract, Keywords])
#3—#1 AND #2
#4—#3 AND (Article AND English [Filter])

Web of Science:

#1—(obesity OR overweight [Topic])
#2—(endurance training OR strength training OR exercise [Topic])
#3—#1 AND #2
#4—#3 AND (Article AND English [Filter])

Moreover, manual searches of the reference lists of included papers were performed to identify further relevant studies and potential studies not captured in the electronic database searches. The research was conducted from database inception to June 2021.

### 2.3. Eligibility Criteria

Original studies were included in this systematic review if they met the following inclusion criteria:

1. Types of studies: randomised trial;
2. Language: articles published in English;
3. Population: free-living adult (≥18 years old) overweight and obese subjects (overweightness, obesity, or one of the following criteria should be listed in the inclusion criteria: body mass index (BMI) ≥25 kg/m^2^ [30] (≥23 kg/m^2^ for Asian populations [31]), waist circumference (WC) ≥80 cm for women and ≥94 cm for men [32], and percentage of fat mass (%FM) >32% for women and 25% for men [33], or equivalent) of either gender and without restrictions based on the ethnicity of study participants, location of study, or sample size;
4. Types of interventions: studies that compare the effects of ET vs. ST training, or/and ET vs. CT, or/and ST vs. CT on inflammatory markers or adipokine levels without any dietary consultation or intervention (study populations should be instructed not to change dietary habits and should not take any dietary supplements), with a duration for the intervention of at least two weeks;
5. Outcomes: only the studies which assessed at least one of the following outcomes were included:
  - Inflammatory parameters and proinflammatory cytokines (1. CRP, 2. IL-6, 3. TNF-α levels);
  - Adipokines (1. leptin, 2. adiponectin, 3. visfatin levels).

The exclusion criteria were as follows:

1. Types of studies: non-randomised trials, uncontrolled trials, observational studies, cohort studies, cross-sectional studies, case-control, case-series, case-report studies, editorial letters, systematic reviews, meta-analyses, conference reports, studies available only as abstracts, and studies with animal models;
2. Language: articles published in any language other than English;
3. Population: children, adolescents, pregnant and breastfeeding women, subjects with rare comorbidities, and subjects living in non-public (closed-type) houses where subjects cannot freely decide on their eating habits or where all residents received the same diet.

### 2.4. Study Selection

**Figure 1:**
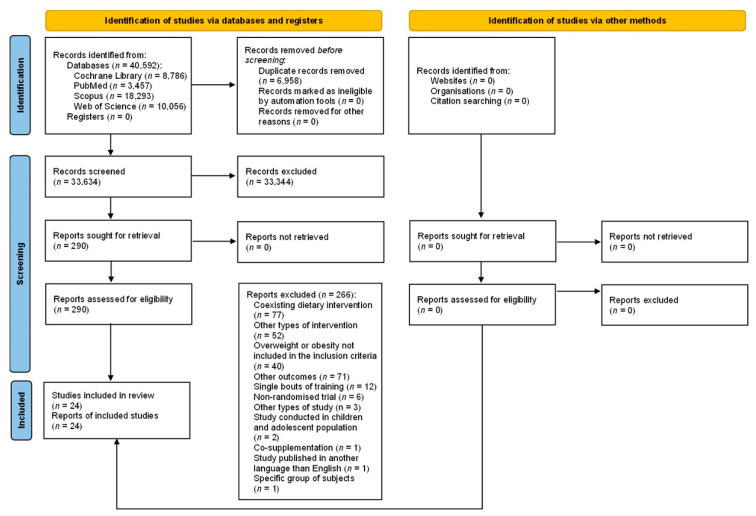
PRISMA 2020 flow diagram.

Two investigators independently evaluated each database (the Cochrane Library: MK and AM, PubMed: MJ and NK, Scopus: MK and AŚ, and Web of Science: NK and AM). All articles were assessed in three main stages of the assessment process (see Figure 1). First, the reviewers screened article titles, then abstracts, and finally full texts for eligibility based on the inclusion and exclusion criteria. Disagreements were resolved by discussion between the reviewers until a consensus was reached. All reviewers agreed on the final decision of studies to be included. With regard to missing data, primary authors were contacted for more information. 

### 2.5. Data Collection Process

A data extraction sheet was developed. Then, the sheet was tested and refined. Two investigators (J.G. and M.J.) independently extracted the data from the included studies and the third investigator (M.K.) checked the extracted data. The fourth investigator (A.M.) converted each outcome to the same units, to facilitate the interpretation of the data. Disagreements were resolved by discussion between the investigators. In the case of missing or unclear information, corresponding authors were contacted by e-mail.

### 2.6. Data Item

The following information was extracted from each included trial:

1. General information: title of the articles, author list, journal name, publication year, country, and continent;
2. Characteristics of the study: study name and design (parallel or cross-over randomised trial) and inclusion and exclusion criteria;
3. Characteristics of the study population: number of subjects who were included, randomised, and completed the trial (total and for each group separately), age, sex, ethnicity, overweight and/or obesity diagnosis methods;
4. Type of intervention: type of training, training volume, training intensity, training frequency, duration of the training, time of intervention, training supervision;
5. Type of outcomes measured: pre-intervention and post-intervention values of each outcome, changes (Δ) for each outcome (post-intervention minus pre-intervention values).

### 2.7. Risk of Bias of Individual Studies

The risk of bias was assessed by two independent investigators (J.G. and M.J.) using the Cochrane risk of bias tool for randomised trials, where the following domains are included: bias due to randomisation, bias due to deviations from intended intervention, bias due to missing data, bias due to outcome measurement, and bias due to selection of reported results [34]. Criteria for low risk, some concerns, and high risk of bias as per the Cochrane handbook for systematic reviews of interventions were used [28].

### 2.8. Statistical Analysis 

The meta-analysis was performed using the Comprehensive Meta-Analysis software, version 3.0 (Biostat, Inc., Englewood, CO, USA). A *p* < 0.05 was considered to be statistically significant. Post-intervention means and standard deviations (SD) were used to perform the meta-analysis. When a standard error was reported, SD was calculated from the standard error of a mean by multiplying by the square root of the sample size. If a 95% confidence interval (CI) was available and the sample size in each group was large (*n* ≥ 100), the SD for each group was obtained by dividing the width of the CI by 3.92, and then multiplying by the square root of the sample size in that group. If the sample size was smaller than 100, the CI was calculated using a value from a t distribution [28]. When the publications revealed median and range, the mean was calculated by the method of Hozo et al. [35]. If logarithmic values were presented, data were transformed back to the raw scale. If a study included at least two groups of the same type of training, but with different intensities, the groups were combined into a single group according to the formula provided in the Cochrane Handbook [28]. The GetData Graph Digitizer software was used to extract data from figures [36]. For studies that reported changes in outcomes >2 time points, the last measurement was used in the meta-analysis. A meta-analysis was carried out on each outcome that was assessed in at least two studies. The original values presented in the publications were used to perform the meta-analysis, while the tables show the values after unifying the units for easier data interpretation. We performed analyses to compare the effect of the following: 1. ET vs. ST; 2. ET vs. CT; and 3. ST vs. CT. Data synthesis was undertaken including a calculation of effect sizes with 95% CI, using fixed-effects models (if no heterogeneity is present), and random-effects models (to analyse outcomes with moderate and high heterogeneity) with inverse variance weighting. Standard mean differences (SMD) of post-intervention values were used as a summary statistic, to allow the comparison of effect sizes across studies. The SMD measures the absolute difference between the mean value in two groups of a trial. Forest plots were generated to illustrate the study-specific effect sizes, along with 95% CI. To determine the presence of publication bias, funnel plots were generated. Additionally, Begg’s and Egger’s tests were performed. Subgroup analyses were not performed. Heterogeneity between studies was evaluated using Cochran Q statistics with *p* < 0.1 indicating significant heterogeneity. The I^2^ test was used to evaluate consistency between studies in which a value <30% indicates a low risk of heterogeneity, 30% to 75% indicates a moderate risk of heterogeneity, and >75% indicates a high risk of heterogeneity, which were consistent with the interpretation thresholds for the I^2^ statistics, according to the Cochrane handbook for systematic reviews (<40% indicates a low risk of heterogeneity, 30% to 60% indicates a moderate risk of heterogeneity, 50 to 90% indicates a significant risk of heterogeneity, and >75% indicates a significant risk of heterogeneity) [28]. Template data collection forms, data extracted from included studies, data used for analysis, analytic code, and any other materials used in the review are available on reasonable request from the corresponding author.

## 3. Results

### 3.1. Search Results 

The search process is presented in Figure 1. A total of 40,592 records were identified, including 6958 duplicate positions that were excluded. Screening of the titles and abstracts excluded a further 33,344 articles and, therefore, 290 full texts were retrieved. Finally, 24 papers were included in the study [14,15,16,17,18,19,20,21,22,23,24,25,37,38,39,40,41,42,43,44,45,46,47,48], of which the two following papers related to the same study conducted on the same population and the same intervention but reported on the different outcomes: Nunes et al. [47] and Martins et al. [48]. Moreover, one manuscript was published in two journals [41,42]. 

### 3.2. Characteristics of Included Studies

The characteristics of the included studies are presented in Table 1. All studies were designed as randomised trials and published between 2005 [45] and 2021 [23]. Most studies were performed in Asia (eight in Iran [17,18,20,37,38,41,42,44,46] two in Japan [19,45], and one in South Korea [24]); six studies were conducted in Europe (two in Poland [14,23], one each in Denmark [21], Finland [40], Greece [15], and Norway [43]); three in South America (Brazil [25,47,48]); and three studies were performed in Australia and Oceania (two in Australia [16,39] and one in New Zealand [22]).

### 3.3. Characteristics of Study Participants

A total of 1145 subjects were included in the studies. The number of subjects participating in each study ranged from 21 [45] to 144 [40], and the number of subjects per group varied from 7 [45] to 52 [23]. All subjects included in the studies were overweight or obese, and were instructed not to change their dietary habits during the intervention period. The following parameters were used to defined excessive body weight: BMI [14,15,17,18,20,23,25,38,39,40,43,45,46,48], WC [14,21,22,23,43], %FM [14,19,23,24,37,47,48], and waist-to-hip ratio (WHR) [21]. The definition of overweightness or obesity was not provided in three studies [16,41,42,44]. In six studies, subjects with DM2 or prediabetes who were overweight or obese were included [22,25,39,40,46,48]. Two studies were conducted in subjects with metabolic syndrome [20,43], and one included subjects with non-alcoholic fatty liver disease [19]. Most of the studies were performed in middle-aged subjects [14,15,16,18,19,20,21,22,23,24,25,38,39,40,43,44,46,47,48], while four studies were conducted with young adults [17,37,41,42,45]. Nine studies were performed in men [16,18,19,20,37,38,40,41,42,45], eight in women [14,17,18,24,29,46,47,48], and six included both men and women [15,21,22,25,39,43]. 

**Table 1:** Characteristics of included studies.

Author	Year	Country (Region)	Groups	*n* Included	*n* Completed	Studied Population	Obesity/Overweight Definition	Age [Years]	Sex [% of Women]
Jamka et al. [23]	2021	Poland (Europe)	ET CT	52 49	44 41	Abdominally obese women	BMI ≥ 30 kg/m^2^ and WC > 80 cm and %FM ≥ 32%	55 ± 7 ^1^ 55 ± 7 ^1^	100
Kang et al. [24]	2020	South Korea (Asia)	ET ST	21 20	21 20	Post-menopausal obese women	%FM > 30%	56.67 ± 5.43 ^1^ 52.50 ± 7.65 ^1^	100
Mohammad Rahimi et al. [20]	2020	Iran (Asia)	ET ST CT CG	10 11 12 11	10 10 10 10	Sedentary obese men with metabolic syndrome	BMI: 30–40 kg/m^2^	44.8 ± 4.8 ^1^ 46.1 ± 5.1 ^1^ 44.9 ± 4.2 ^1^ 46.4 ± 5.1 ^1^	0
Banitalebi et al. [46]	2019	Iran (Asia)	ET CT CG	17 17 18	14 14 14	Overweight or obese women with T2DM	BMI: 25–48 kg/m^2^	55.36 ± 5.94 ^1^ 54.14 ± 5.43 ^1^ 55.71 ± 6.40 ^1^	100
Christensen et al. [21]	2019	Denmark (Europe)	ET ST CG	16 16 18	14 13 12	Inactive subjects with abdominal obesity	WHR ≥ 0.5 and/or WC ≥ 88 cm for women or WC ≥ 102 cm for men	39 ± 14 ^1^ 38 ± 14 ^1^ 47 ± 12 ^1^	75
Nunes et al. [47] ^a^	2019	Brazil (South America)	ET CT	13 13	13 13	Obese postmenopausal women with no history of physical training practice	%FM > 40%	62.3 (58.2–66.5) ^2^ 62.9 (57.6–68.2) ^2^	100
Ratajczak et al. [14]	2019	Poland (Europe)	ET CT	22 22	22 17	Women with simple obesity	BMI ≥ 30 kg/m^2^ and WC > 80 cm and %FM ≥ 33%	51 ± 8 ^1^ 49 ± 10 ^1^	100
Martins et al. [48] ^a^	2018	Brazil (South America)	ET CT	14 14	8 8	Overweight women with high risk for TDM2, no exercising for at least 6 months	BMI > 24.9 kg/m^2^ and %FM > 40%	64.3 ± 6.7 ^1^ 65.0 ± 6.3 ^1^	100
Oh et al. [19]	2017	Japan (Asia)	ET ^3^ ET ^4^ ST	21 19 20	20 13 19	Obese sedentary men with nonalcoholic fatty liver disease and no exercise habits	%FM > 25%	48.2 ± 2.3 ^5^ 48.6 ± 1.8 ^5^ 51.2 ± 1.9 ^5^	0
Soori et al. [18]	2017	Iran (Asia)	ET ST CT CG	8 8 8 8	NI	Postmenopausal sedentary obese women	BMI ≥ 30 kg/m^2^	45–60 ^6,7^	100
Shahram et al. [17]	2016	Iran (Asia)	ET ST CG	30	NI	Sedentary young study women	BMI ≥ 25 kg/m^2^	22.4 ± 1.64 ^1^ 22.3 ± 1.41 ^1^ 22.77 ± 1.63 ^1^	100
Tayebi et al. [37]	2016	Iran (Asia)	ET ST CT	12 12 12	11 9 12	Non-athlete men with obesity	%FM > 25%	21.48 ± 1.46 ^1,8^	0
Nikseresht et al. [38]	2014	Iran (Asia)	ET ST CG	12 12 10	NI	Sedentary overweight or obese men with no regular exercise with no history of any medical condition	BMI > 25 kg/m^2^	39.6 ± 3.7 ^1^ 40.4 ± 5.2 ^1^ 38.9 ± 4.1 ^1^	0
Donges et al. [16]	2013	Australia (Australia)	ET ST CT CG	13 13 13 8	13 13 13 8	Sedentary overweight middle-aged men	NI	45.4 ± 1.7 ^5^ 51.7 ± 2.1 ^5^ 46.2 ± 1.4 ^5^ 49.5 ± 2.6 ^5^	0
Ho et al. [39]	2013	Australia (Australia)	ET ST CT CG	19 22 20 19	15 16 17 16	Sedentary to lightly active overweight or obese subjects	BMI ≥ 25 and ≤40 kg/m^2^	55 ^8^ (44–62) ^6^ 52 ^8^ (43–59) ^6^ 53 ^8^ (43–64) ^6^ 52 ^8^ (40–66) ^6^	84
Kadoglou et al. [15]	2013	Greece (Europe)	ET ST CT CG	25 25 25 25	21 23 22 24	Overweight or obese subjects with T2DM	BMI ≥ 25 kg/m^2^	58.3 ± 5.4 ^1^ 56.1 ± 5.3 ^1^ 57.9 ± 6.5 ^1^ 57.9 ± 7.2 ^1^	72
Venojärvi et al. [40]	2013	Finland (Europe)	ET ST CG	48 49 47	39 36 40	Overweight and obese middle-aged men with impaired glucose tolerance	BMI: 25.1–34.9 kg/m^2^	55 ± 6.2 ^1^ 54 ± 6.1 ^1^ 54 ± 7.2 ^1^	0
Asad et al. [41,42]	2012	Iran (Asia)	ET ST CT CG	12 9 13 10	12 9 13 10	Sedentary healthy male college students	NI	22 ± 0.89 ^1^ 21 ± 1.57 ^1^ 21.38 ± 2.6 ^1^ 21.44 ± 1.13 ^1^	0
Stensvold et al. [43]	2012	Norway (Europe)	ET ST CG	11 11 11	11 10 10	Inactive subjects with metabolic syndrome	BMI ≥ 30 kg/m^2^ or WC ≥ 80 cm for women or WC ≥ 94 cm for men	49.9 ± 10.1 ^1^ 50.9 ± 7.6 ^1^ 47.3 ± 10.2 ^1^	23
Sukala et al. [22]	2012	New Zealand (Australia)	ET ST	13 13	9 9	Subjects with T2DM and visceral obesity	WC ≥ 88 cm for women or WC ≥ 102 cm for men	51 ± 4 ^1^ 48 ± 6 ^1^	72
Jorge et al. [25]	2011	Brazil (South America)	ET ST CT CG	12 12 12 12	NI ^8^	Overweight or obese subjects with T2DM	BMI: 25–40 kg/m^2^	52.09 ± 8.71 ^1^ 54.1 ± 8.94 ^1^ 57.90 ± 8.06 ^1^ 53.42 ± 9.82 ^1^	62
Ahmadizad et al. [44]	2007	Iran (Asia)	ET ST CG	8 8 8	NI	Sedentary obese healthy men	NI	41.3 ± 5.1 ^1^ 40.9 ± 3.2 ^1^ 38.6 ± 3.2 ^1^	0
Hara et al. [45]	2005	Japan (Asia)	ET CT CG	7 7 7	7 7 7	Young obese men	BMI > 25 kg/m^2^	19.7 ± 1.3 ^1^ 18.4 ± 0.5 ^1^ 19.4 ± 1.0 ^1^	0

BMI—body mass index; CG—control group; CT—combined training; ET—endurance training; NI—no information; ST—strength training; T2DM—type 2 diabetes mellitus; WC—waist circumference; WHR—waist to hip ratio; %FM—percentage of fat mass. ^1^ Mean ± standard deviation; ^2^ Mean and 95% confidence interval; ^3^ High-intensity interval training; ^4^ Moderate-intensity continuous training; ^5^ Mean ± standard error; ^6^ Range; ^7^ Data for the total population; ^8^ Mean; ^a^ Studies marked with the same letters were conducted in the same population.

### 3.4. Characteristics of the Study Intervention

Characteristics of training programmes are presented in Table 2. ET was evaluated by 23 studies [14,15,16,17,18,19,20,21,22,23,24,25,37,38,39,40,41,42,43,44,45,46,47,48], 17 papers assessed ST [15,16,17,18,19,20,21,22,24,25,37,38,39,40,41,42,43,44], and 15 studies evaluated CT [14,15,16,18,20,23,25,37,39,41,42,43,45,46,47,48]. Additionally, 15 studies also included a control group [15,16,17,18,20,21,25,38,39,40,41,42,43,44,45,46]. In most studies, the control group did not receive physical activity intervention and was instructed to maintain their usual physical activity level [16,17,18,20,39,40,41,42,43,44,45,46]. In one study, subjects were encouraged to perform self-controlled, leisure-time physical activity [15], and one study had subjects from the control group perform light stretching exercises [25]. In twelve studies, the intensity of ET was measured using heart rate (HR) max [14,15,16,18,23,37,38,41,42,44,46,47,48], in four studies, the intensity of ET was expressed in HR reserve [22,24,39,40] and two studies presented the intensity of training in HR peak [20,43] and oxygen uptake (VO_2_ max) [19,45]. In one study, the intensity of ET was expressed as target HR [17], and, in one, as HR corresponding to the lactate threshold [25]. Moreover, in one study the intensity of ET was not reported [21]. In 18 studies, the intensity of ST was measured in repetition maximum (RM) [14,15,16,17,18,19,20,21,23,24,37,38,40,43,44,45,47,48], and five studies provided no information about the intensity of the ST [22,25,39,41,42,46]. The duration of ET varied between 13 [19] and 60 min [14,15,16,22,23,25,40]. In one study the duration of ET was not provided [17]. The duration of ST ranged from 30 [39] to 68 min [47,48]. In five studies, the duration of ST was not reported [16,17,19,37,41,42]. Mixed training duration ranged from 30 [39] to 90 min [20,43,45]. In two studies, the duration of CT was not reported [16,41,42]. In seven studies, the duration of CT was similar to the duration of ET and/or ST [14,15,18,23,25,39,46], while five studies involved mixed training of longer duration than the ET or ST alone [20,43,45,47,48]. In most studies, training was performed three times per week [14,16,17,18,19,20,21,22,23,24,25,37,38,40,41,42,43,44,46,47,48]. In one study, training was performed four times per week [15], and, in one study, five times per week [39]. Moreover, in one study, ET was performed three times per week, while the combined group performed ST two or three times per week together with the ET three times per week [45]. The duration of the intervention period ranged from 8 [37,41,42,45] to 26 weeks [15]. In most of the studies, training programmes were supervised [14,15,16,18,19,20,21,22,23,24,25,38,40,43,44,46,47,48], except one study [39]. Moreover, four studies did not provide information about the supervision of training [17,37,41,42,45].

### 3.5. Effect of Physical Training on CRP Concentrations 

The effects of the training interventions on inflammatory markers are presented in Table 3. Ten studies compared the effect of different training programmes on CRP levels [14,15,16,21,22,23,25,40,43,48]: three studies evaluated the effect of ET and ST [21,22,40], three assessed the effect of ET and CT [14,23,48], and four evaluated the effect of ET, ST, and CT [15,16,25,43]. However, only one study reported significant differences between the effect of ET and ST, as well as between CT and ST [15].

**Table 2:** Characteristics of training programmes.

Author	Year	Groups	Characteristic of Groups/Training (Including Volume)	Intensity of Training [%]	Duration of Training [Min]	Frequency of Training [Days per Week]	Time of Intervention [Weeks]	Supervision
Jamka et al. [23]	2021	ET	Cycling on ergometer	50–70% of HR max	60	3	12	Yes
		CT	ET: Cycling on ergometer ST: Exercises with a barbell and a gymnastic ball; the goal number of repetitions per set was 16 in barbell curls and 30 in barbell squats; between the series, 10–15 s pauses were taken	ET: 50–70% of HR max ST: 50–60% of 1 RM				
Kang et al. [24]	2020	ET	Endurance exercise performed on a treadmill	50–60% of HR reserve	50	3	12	Yes
		ST	3 sets of 7 exercises with a 1 min rest between sets and a 1 min rest between the different exercises: squat and lunge for the lower body, chest press and vertical fly for the chest, lat pull-downs and long pulls for the back, and crunches for the abdomen	55–65% of 1 RM	60			
Mohammad Rahimi et al. [20]	2020	ET	4 × 4 min intervals of walking/running on a treadmill, with 3 min exercise between each interval	90% of HR peak (intervals) 70% of HR peak (between intervals)	43	3	12	Yes
		ST	2–3 sets of 7 weight machine exercises of 8–20 repetitions: lateral pull-down, chest press, seated row, triceps push-down, knee flexion, knee extension, and leg press	40–80% of 1 RM ^1^	45			
		CT	Exercises were similar to the practices of the other two groups	ET: 90% of HR peak (intervals) 70% of HR peak (between intervals) ST: 40–80% of 1 RM ^1^	ET: 43 ST: 45			
		CG	The group was advised not to change their physical activity levels throughout the intervention	N/A	N/A	N/A		
Banitalebi et al. [46]	2019	ET	Sprint interval training performed on cycle ergometers at a pedaling rate of 20 rpm	60–70% of HR max ^1^	50 ^1^	3	10	Yes
		CT	ET: Treadmill or cycle ergometer ST: 1–3 set of 5 exercises of 10–15 repetitions with 10–15 RM ^1^ and 2–3 min rest between sets ^1^; training was performed on weight stack machines and included bilateral leg press, lateral pull-down, bench press, bilateral biceps curl, and bilateral triceps push down	ET: 60–70% of HR max ^1^ ST:NI				
		CG	Continued their usual medical care and received diabetes recommendations for self-management	N/A	N/A	N/A		
Christensen et al. [21]	2019	ET	High intensive interval exercise performed on an ergometer bicycle	NI	45	3	12	Yes
		ST	3–5 sets of 10 exercises	60–80% of 1 RM ^1^				
		CG	No intervention	N/A	N/A	N/A		
Nunes et al. [47] ^a^ Martins et al. [48] ^a^	2019 2018	ET	High-intensity interval bodyweight training; 10 sets of vigorous exercises (30 s of stair climbing and 30 s of body weight squats) interspersed by 60 s of a light walk ^2^	>85% of HR max + recovery at 60–70% of HR max	36	3	12	Yes
		CT	ET: Moderate walking ST: 1–3 sets of 5 strength exercises of 8–12 repetitions ^1^ with 1.5 min rest intervals between the sets and exercises (half squat, bench press, leg curl, rowing machine, and unilateral leg extension)	ET: 70% of HR max ST: 70% of 1 RM ^2^	68 (including 30 min of ET^2^)			
Ratajczak et al. [14]	2019	ET	Training on cycle ergometers	60–80% of HR max	60	3	13	Yes
		CT	ET: Similar as described for ET ST: Exercises using a neck barbell and gymnastics ball; on Mondays, upper limb exercises were performed with a neck barbell; Wednesdays involved spine-stabilizing exercises, deep muscle-forming exercises, and balance-adjusting exercises with a gymnastic ball; on Fridays, lower limb exercises with a neck barbell were carried out; the number of repetitions was systematically increased with the increase in subject’s muscle strength	ET: 60–80% of HR max ST: 50–60% of 1 RM	60 (ET: 25 + ST: 20 + warm up: 5 + cool down: 10)			
Oh et al. [19]	2017	ET ^3^	3 sets of 3 min cycling with a 2 min active rest between sets, energy expenditure: 180 kcal	80–85% of VO_2_ max (rest at 50% of VO_2_ max)	13	3	12	Yes
		ET ^4^	Cycling, energy expenditure: 360 kcal	60–65% of VO_2_ max	40			
		ST	Consisted of sit-ups, leg presses, leg extensions, leg curls, chest presses, seated rows, and pull-down, energy expenditure: 180 kcal	To 60% of 1 RM for lower body exercises 30–60% of 1 RM for upper body exercises	NI			
Soori et al. [18]	2017	ET	Water-based training: swimming or walking in the water	40–60% of HR max ^1^	45	3	10	Yes
		ST	3 sets of 6 dynamic exercises with free weights of 10–12 repetitions: bench press, lateral pull-down, rowing, leg press, and hip flexion and extension	40–60% of 1 RM ^1^				
		CT	ET: Swimming ST: 2 sets of 10–12 repetitions of resistance exercises described in the ST group	ET: 40–60% of HR max ST: 40–60% of 1 RM ^1^	44 (ET: 22 + ST: 22)			
		CG	No intervention	N/A	N/A	N/A		
Shahram et al. [17]	2016	ET	Continues raining, distance:1600–3200 m	60–75% of target HR	NI	3	12	NI
		ST	Circuit weight training in 11 stations and included 4 sets with 12 RM	50–60% of 1 RM				
		CG	No intervention	N/A				
Tayebi et al. [37]	2016	ET	Running program	65–85% of HR max ^1^	25–40 ^1^	3	8	NI
		ST	6 sets of 5 exercises of 3–12 repetitions: leg press, knee extension, lat pulldown, biceps curls, and dead lift	50–80% of 1 RM	NI			
		CT	ET: Similar as described for ET ST: 3 sets of 5 listed in the ST group exercises, 4–12 repetitions	ET: 65–85% of HR max ^1^ ST: 50–80% of 1 RM				
Nikseresht et al. [38]	2014	ET	Running on a treadmill; 4 sets of 4 min with 3 min recovery intervals	80–90% of HR max (recovery intervals at 55–65% of HR max)	25 ^5^	3	12	Yes
		ST	1–4 sets of 12 exercises of 2–20 repetitions with 1–7 min of rest period: knee extension, bench press, incline bench press, seated row, dead lift, pulley crunches, lat pull-downs, calf raise, hamstring curl, press behind neck, upright row, and arm curl	40–95% of 1 RM	40–65			
		CG	Continued their normal sedentary life	N/A	N/A	N/A		
Donges et al. [16]	2013	ET	Cycling with elliptical cross training	75–80% of HR max	40–60 ^1^	3	12	Yes
		ST	Whole-body training program, including chest and shoulder press, seated rows, lat pulldown, leg press, leg curls, lunges, machine squats, and deadlifts; 3–4 sets × 8–10 of each exercise	75–80% of 1 RM ^1^	NI			
		CT	ET: Similar as described for ET ST: 1.5–2 × 8–10 of each exercise described in the ST group	ET: 75–80% of HR max ST: 75–80% of 1 RM ^1^	ET: 20–30 ST:NI			
		CG	No intervention	N/A	N/A	N/A		
Ho et al. [39]	2013	ET	Treadmill walking	60% of HR reserve ± 10 beats/min	30	5	12	No
		ST	4 sets of 5 exercises of 8–12 repetitions at 10 RM of leg press, leg curl, leg extension, bench press, and rear deltoid row	NI				
		CT	ET: Similar as described for ET ST: 2 sets of 8–12 repetitions at 10 RM of exercises described in the ST group	ET: 60% of HR reserve ± 10 beats/min ST: NI	30 (ET: 15 + ST: 15)			
		CG	No exercise; subjects were requested to continue their normal physical activity and received a placebo dietary supplement only	N/A	N/A	N/A		
Kadoglou et al. [15]	2013	ET	Walking or running on a treadmill, cycling or calisthenics	60–75% of HR max	60	4	26	Yes
		ST	2–3 sets of 8 types of exercises of 8–10 repetitions: seated leg press, knee extension, knee flexion, chest press, lat pulldown, overhead press, biceps curl, and triceps extension	60–80% of 1 RM	60 ^1,2^			
		CT	CT: combined training as in endurance training group and strength training group with following pattern weekly: 1 session of ET programme; 1 session of ST; and 2 sessions combining the types of exercise of both ET and ST in the same session	ET: 60–75% of HR max ST: 60–80% of 1 RM	55 ^1,2^			
		CG	Subjects were encouraged to perform self-controlled, leisure-time physical activity (e.g., walking briskly, cycling outdoors)	Low-to-moderate intensity	150/week	N/A		
Venojärvi et al. [40]	2013	ET	Nordic walking consisted of warm-up exercises including walking for 5 min and stretching of main muscle groups in addition to walking with poles; after the pole walking, the main muscle groups were stretched for 5 min for cool-down	55–75% of HR reserve ^1^	60	3	12	Yes
		ST	Started with warm-up exercises including cycling or rowing with ergometer for 5 min and stretching of main muscle groups. After that the main part of programme was performed by using regular strength equipment, and training focus was on strength and power exercises of the lower extremities and trunk; muscles of the upper extremities were also trained. Muscle contractions were performed with maximal or high velocity, and external loads were 50–85% from exercise-specific maximal strength, which was determined by the 5RM; At the end of every session, subjects cooled down by cycling or rowing with the ergometer for 5 min and by stretching the main muscle groups	50–85% from exercise-specific maximal strength, which was determined by the 5 RM				
		CG	No intervention	N/A	N/A	N/A		
Asad et al. [41,42]	2012	ET	Running program	65–85% of HR max ^1^	25–40 ^1^	3	8	NI
		ST	3 sets of 10–15 repetitions ^1^ of weight training exercise with machines and free loads; the training program contained upper body training and lower body training, such as: bench press, sitting and standing up with halter, leg extension, leg flexion, and leg press, and rowing	NI	NI			
		CT	Trained the sum of ET and ST groups	ET: 65–85% of HR max ^1^ ST:NI	ET: 25–40 ^1^ + ST:NI			
		CG	No intervention	N/A	N/A	N/A		
Stensvold et al. [43]	2012	ET	Endurance interval training: treadmill walking or running (self-selected); consisted of 4 intervals of 4 min at and 3 min active recovery period	Intervals: 90–95% of HR peak Recovery period: 70% of HR peak	43	3	12	Yes
		ST	3 sets of 8–12 repetitions; consisted of two different programmes including different muscle groups; the following exercises were performed twice weekly (program 1): low row, bench press, and hack lift; the alternative program was performed once each week (program 2): deltoid exercise (lateral raise exercise), triceps pulldown, biceps curl, and low-row and core exercises (plank exercise)	60–80% of 1 RM ^1^	40–50 ^6^			
		CT	ET twice a week and ST once a week	ET: 90–95% of HR peak ST: 60–80% of 1 RM ^1^	ET: 43 ST: 40–50 ^6^			
		CG	No intervention	N/A	N/A	N/A		
Sukala et al. [22]	2012	ET	Exercises on a cycle ergometer	65–85% of HR reserve ^1^	40–60 ^1^	3	16	Yes
		ST	2–3 sets of 8 exercises of 6–8 repetition with 1 min rest between sets and exercises; exercises were performed using machine weights targeting all the major muscle groups of the body and included: seated leg press, knee extension, knee flexion, chest press, lat pulldown, overhead press, biceps curl, and triceps extension	NI				
Jorge et al. [25]	2011	ET	Cycling.programme	HR corresponding to the lactate threshold	60	3	12	Yes
		ST	Focused on the large muscle groups and consisted of a 7-exercise circuit as follows: leg press, bench press, lat pull down, seated rowing, shoulder press, abdominal curls, and knee curls	NI				
		CT	Consisted of ST interchanged with ET performed at the same intensity and half the volume of the ET and ST groups	ET: HR corresponding to the lactate threshold ST:NI				
		CG	Light stretching exercises	N/A	N/A			
Ahmadizad et al. [44]	2007	ET	Continuous running	75–85% of HR max	20–30 ^1^	3	12	Yes
		ST	4 sets of circuit weight training for 11 stations; the maximum numbers of repetitions in each station was 12; exercises involving the upper and lower body	50–60% of 1 RM	50–60			
		CG	No intervention	N/A	N/A	N/A		
Hara et al. [45]	2005	ET	Training on treadmills and cycle ergometers	40.8–54.8% of VO_2_ max	30–45	3	8	NI
		CT	ET: Similar as described for ET ST: Included the following types of exercise: arm curl, triceps extension, and shoulder press for upper-limb training; squat, leg press, leg curl, leg extension, and calf raise for lower-limb training; and bench press, seated butterfly, lat pull-down, trunk curl, back extension, and dead lift for trunk training. Participants selected 2 types each from the upper and lower limb training options, and 3 from trunk training choices, and thus performed 7 exercises in each training session; 3 sets for each exercise consisting of 10 repetitions	ET: 40.8–54.8% of VO_2_ max ST: 80% of 1 RM	80–90 (ET: 30 + ST: 50–60)	ET: 3 + ST: 2–3	22	
		CG	No intervention	N/A	N/A	N/A	NI	

CG—control group; CT—combined training; ET—endurance training; HR—heart ratio; N/A—not applicable; NI—no information; RM—repetition maximum; ST—strength training; VO_2_—oxygen uptake. ^1^ Increasing progressively over time; ^2^ The goal duration/volume of training; ^3^ High-intensity interval training; ^4^ Moderate-intensity continuous training; ^5^ Four sets of 4 min training with 3 min recovery; ^6^ Programme 1:40 min, programme 2:50 min; ^a^ Studies marked with the same letters were conducted in the same population.

**Table 3:** Inflammatory parameters levels in studied populations.

Author	Year	Group	CRP [mg/L]			IL-6 [pg/mL]			TNF-α [pg/mL]		
			Pre	Post	Changes	Pre	Post	Changes	Pre	Post	Changes
Jamka et al. [23]	2021	ET	3.87 ± 3.76 ^2^	4.34 ± 4.63 ^2^	0.47 ± 2.86 ^2^	2.19 ± 1.54 ^2^	2.34 ± 1.60 ^2^	0.16 ± 1.79 ^2^	40 ± 52 ^2^	36 ± 48 ^2^	−3 ± 12 ^2^
		CT	3.95 ± 3.85 ^2^	4.22 ± 4.29 ^2^	0.27 ± 2.99 ^2^	1.78 ± 0.91 ^2^	2.16 ± 1.14 ^2^	0.39 ± 1.11 ^2^	38 ± 49 ^2^	40 ± 51 ^2^	2 ± 16 ^2^
		*p * ^1^				CT: *p* = 0.03 (pre vs.post)			ET: *p* = 0.003 (pre vs. post)		
Kang et al. [24]	2020	ET	NI	NI		20.11 ± 2.6 ^2,3^	22.34 ± 3.2 ^2,3,4^ 24.57 ± 4.1 ^2,3,5^	NI	NI	NI	NI
		ST				22.15 ± 5.4 ^2,3^	24.2 ± 6.9 ^2,3,4^ 27.37 ± 5.6 ^2,3,5^				
		*p * ^1^				ET: *p* < 0.01, ST: *p* < 0.05 (pre vs. middle) ET, ST: *p* < 0.001 (pre vs. post) ET, ST: *p* < 0.01 (middle vs. post)					
Banitalebi et al. [46]	2019	ET	NI	NI	NI	1.89 ± 0.95 ^2^	1.21 ± 1.11 ^2^	−0.67	NI	NI	NI
		CT				2.03 ± 1.08 ^2^	1.50 ± 1.32 ^2^	−0.52			
		CG				2.12 ± 1.24 ^2^	1.88 ± 2.01 ^2^	−0.23			
		*p * ^1^				*p* = 0.002 (time) *p* = 0.009 (group × time)					
Christensen et al. [21]	2019	ET	NI	33.6 (1.05–66.15) ^6^	0.0 (−32.55–32.55) ^6^ 3 (−76–83)% ^6,7^	NI	0.7 (0.4–1.1) ^6^	0.0 (−0.4–0.3) ^6^ −2 (−52–49)% ^6,7^	NI	2.4 (2.0–2.8) ^6^	0.1 (−0.3–0.5) ^6^ 5.8 (−6.5–18.1)% ^6,7^
		ST		61.95 (28.35–96.6) ^6^	28.35 (−5.25–63) ^6^ 64 (−19–147)% ^6,7^		1.1 (0.7–1.5) ^6^	0.4 (0.0–0.8) ^6^ 70 (16–124)% ^6,7^		2.5 (2.0–2.9) ^6^	0.2 (−0.3–0.6) ^6^ 7.7 (−5.1–20.6)% ^6,7^
		CG		32.55 (−4.2–69.3) ^6^	−1.05 (−37.8–35.7) ^6^ 8 (−82–98)% ^6,7^		0.8 (0.4–1.2) ^6^	0.1 (−0.3–0.5) ^6^ 30 (−26–87)% ^6,7^		2.4 (1.9–2.9) ^6^	0.1 (−0.4–0.6) ^6^ 4.8 (−9.2–18.9)% ^6,7^
Nunes et al. [47] ^a^	2019	ET	NI	NI	NI	1.4 (0.7–2.0) ^8^	2.6 (1.4–3.9) ^8^	1.2 (0.4–2.1) ^8^ 85.7 (28.6–149.9)% ^7,8,9^	NI	NI	NI
		CT				1.8 (0.6–3.1) ^8^	1.7 (1.0–2.4) ^8^	−0.1 (−1.1–0.9) ^8^ −5.6 (−61.1–50.4)% ^7,8,9^			
		*p * ^1^				*p* = 0.037 (time × group)					
Ratajczak et al. [14]	2019	ET	4.18 ± 2.50 ^2^	3.45 ± 2.50 ^2^	NI	NI	NI	NI	NI	NI	NI
		CT	3.49 ± 3.20 ^2^	2.52 ± 1.90 ^2^							
		*p * ^1^	CT: *p* < 0.05 (pre vs. post)								
Martins et al. [48] ^a^	2018	ET	0.5 ± 0.5 ^2^	0.7 ± 0.6 ^2^	40.0% ^7,9^	1.4 ± 1.1 ^2^	2.6 ± 2.2 ^2^	85.7% ^7,9^	NI	NI	NI
		CT	0.1 ± 0.1 ^2^	0.1 ± 0.1 ^2^	0% ^7,9^	1.3 ± 1.8 ^2^	1.2 ± 1.1 ^2^	−7.7% ^7,9^			
Oh et al. [19]	2017	ET ^10^	NI	NI	NI	NI	NI	−0.35	NI	NI	−0.068 ^12^
		ET ^11^						1.06			0.003 ^12^
		ST						0.63			0.092 ^12^
Shahram et al. [17]	2016	ET	NI	NI	NI	7.16 ± 0.15 ^2^	2.71 ± 0.14 ^2^	NI	12.31 ± 0.23 ^2^	9.16 ± 0.19 ^2^	NI
		ST				7.10 ± 0.21 ^2^	2.84 ± 0.34 ^2^		12.25 ± 0.27 ^2^	9.21 ± 0.24 ^2^	
		CG				7.19 ± 0.15 ^2^	7.26 ± 0.13 ^2^		12.80 ± 0.24 ^2^	12.06 ± 0.25 ^2^	
		*p * ^1^				*p* < 0.05 (pre vs. post)			*p* < 0.05 (pre vs. post)		
Nikseresht et al. [38]	2014	ET	NI	NI	NI	NI	NI	NI	2.99 ± 0.64 ^2^	2.60 ± 0.54 ^2^	−11.9% ^7^
		ST							3.00 ± 0.46 ^2^	2.66 ± 0.53 ^2^	−10.7% ^7^
		CG							2.90 ± 0.74 ^2^	2.96 ± 0.64 ^2^	NI
		*p * ^1^							ET: *p* = 0.01 (pre vs. post, changes) ST: *p* = 0.04 (pre vs. post, changes) *p* = 0.025 (time)		
Donges et al. [16]	2013	ET	2.25 ± 0.37 ^13^	2.33 ± 0.21 ^13^	3 ± 13% ^7,13^	1.94 ± 0.31 ^13^	1.28 ± 0.26 ^13^	–34 ± 11% ^7,13^	4.42 ± 0.33 ^13^	3.29 ± 0.29 ^13^	−26 ± 10% ^7,13^
		ST	2.21 ± 0.30 ^13^	2.38 ± 0.31 ^13^	8 ± 9% ^7,13^	2.74 ± 0.69 ^13^	1.84 ± 0.53 ^13^	–33 ± 18% ^7,13^	7.14 ± 0.43 ^13^	6.23 ± 0.32 ^13^	−12 ± 5% ^7,13^
		CT	1.88 ± 0.27 ^13^	1.91 ± 0.34 ^13^	1 ± 14% ^7,13^	2.35 ± 0.31 ^13^	1.91 ± 0.26 ^13^	–19 ± 6% ^7,13^	5.21 ± 0.66 ^13^	4.39 ± 0.41 ^13^	−16 ± 10% ^7,13^
		CG	1.60 ± 0.09 ^13^	1.89 ± 0.32 ^13^	18 ± 19% ^7,13^	1.93 ± 0.60 ^13^	1.88 ± 0.94 ^13^	–3 ± 19% ^7,13^	6.11 ± 0.25 ^13^	6.19 ± 0.33 ^13^	1 ± 7% ^7,13^
		*p * ^1^				ET, ST, CT: *p* < 0.05 (pre vs. post)			ET, ST, CT: *p* < 0.05 (pre vs. post) ET vs. ST, CG: *p* < 0.05 (pre, post-hoc)		
Ho et al. [39]	2013	ET	NI	NI	NI	2.5 (0.0–8.5) ^14^	NI	NI	14.6 (8.1–23.3) ^14^	NI	−20.8% ^7^
		ST				2.3 (0.0–7.4) ^14^			12.0 (6.4–20.0) ^14^		−26.9% ^7^
		CT				2.3 (0.0–12.4) ^14^			12.6 (4.3–25.8) ^14^		−32.6% ^7^
		CG				3.0 (0.0–13.1) ^14^			10.2 (4.9–17.0) ^14^		NI
		*p * ^1^							ET: *p* = 0.011, ST: *p* = 0.0001, CT: *p* = 0.003 (pre vs. post) CT vs. CG: *p* = 0.025 ^15^ (changes, post-hoc)		
Kadoglou et al. [15]	2013	ET	0.15 ± 0.04 ^2,16^	NI	−0.05 ± 0.01 ^2,16^ −33.3 ± 6.7% ^7,9,2^	NI	NI	NI	NI	NI	NI
		ST	0.15 ± 0.03 ^2,16^		0.011 ± 0.003 ^2,16^ 7.3 ± 2% ^2,7^						
		CT	0.14 ± 0.05 ^2,16^		−0.05 ± 0.009 ^2,16^ −35.7 ± 6.4% ^2,7,9^						
		CG	0.15 ± 0.04 ^2,16^		0.01 ± 0.02 ^2,16^ 6.7 ± 13.3% ^2,7,9^						
		*p * ^1^	*p* < 0.001 (changes) ET, CT: *p* < 0.05 (pre vs. post) CT, ET vs. ST; ET vs. CG: *p* < 0.001, CT vs. CG: *p* = 0.003 (change, post-hoc)								
Venojärvi et al. [40]	2013	ET	2.2 ± 0.4 ^13,16^	NI	−0.5 ± 0.4 ^13,16^	11.5 ± 3.3 ^13^	NI	−0.4 ± 0.9 ^13^	5.6 ± 0.4 ^13^	NI	−0.2 ± 0.3 ^13^
		ST	1.6 ± 0.3 ^13,16^		0.3 ± 0.4 ^13,16^	7.6 ± 2.3 ^13^		0.3 ± 0.5 ^13^	5.5 ± 0.8 ^13^		−0.2 ± 0.4 ^13^
		CG	1.4 ± 0.2 ^13,16^		−0.1 ± 0.3 ^13,16^	4.0 ± 1.1 ^13^		0.7 ± 0.8 ^13^	4.6 ± 0.3 ^13^		0.5 ± 0.2 ^13^
		*p * ^1^	*p* = 0.050 (change)			*p* = 0.016 (pre) CG vs. ET: *p* = 0.015 ^17^ (pre, post-hoc)					
Stensvold et al. [43]	2012	ET	NI	NI	−0.10 (1.15–−4.71) ^3,18^	NI	NI	0.2 (−3.5–0.8) ^3,18^	NI	NI	−0.13 (0.53–(−1.16)) ^18^
		ST			0.37 (2.38–(−1.72)) ^3,18^			0.3 (−5.4–7.2) ^3,18^	3.9 ± 0.8 ^2^	4.3 ± 0.9 ^2^	0.40 (1.04–(−0.15)) ^18^
		CG			0.59 (1.43–(−4.51)) ^3,18^			0.1 (−0.3–0.8) ^3,18^	NI	NI	0.49 (1.09–(−0.99)) ^18^
		*p * ^1^							ST: *p* = 0.014 (pre vs. post) ET vs. ST: *p* = 0.032, ET vs. CG: *p* = 0.039 (post, post-hoc)		
Sukala et al. [22]	2012	ET	0.8 ± 0.4 ^2,12^	0.6 ± 0.4 ^2,12^	−0.2 ± 0.4 ^2,12^	NI	NI	NI	NI	NI	NI
		ST	0.6 ± 0.5 ^2,12^	0.5 ± 0.5 ^2,12^	−0.2 ± 0.5 ^2,12^						
Jorge et al. [25]	2011	ET	14.35 ± 4.51 ^2,16^	12.95 ± 3.41 ^2,16^	NI	21.15 ± 1.44 ^2^	21.06 ± 1.36 ^2^	NI	2.38 ± 1.31 ^2^	2.46 ± 1.26 ^2^	NI
		ST	16.55 ± 2.55 ^2,16^	14.39 ± 1.80 ^2,16^		21.39 ± 2.60 ^2^	26.11 ± 18.43 ^2^		2.91 ± 2.44 ^2^	4.76 ± 5.18 ^2^	
		CT	15.64 ± 3.86 ^2,16^	14.14 ± 2.56 ^2,16^		20.93 ± 0.86 ^2^	20.23 ± 0.83 ^2^		3.47 ± 1.40 ^2^	3.10 ± 1.08 ^2^	
		CG	15.05 ± 4.22 ^2,16^	12.24 ± 4.31 ^2,16^		23.69 ± 9.81 ^2^	21.29 ± 0.91 ^2^		2.29 ± 0.46 ^2^	2.74 ± 1.10 ^2^	
		*p * ^1^	*p* < 0.05 (pre vs. post)								

CG—control group; CRP—C-reactive protein; CT—combined training; ET—endurance training; IL-6—interleukin 6; N/A—not applicable; NI—no information; post—after intervention; pre—before intervention; ST—strength training; TNF-α—tumor necrosis factor; ^1^ Only statistically significant values are shown; ^2^ Mean ± standard deviation; ^3^ Data from figure; ^4^ 6th week of intervention; ^5^ 12th week of intervention; ^6^ Least square means (means adjusted for baseline) with (95% confidence intervals); ^7^ Relative changes; ^8^ Mean and 95% confidence intervals; ^9^ Converted values; ^10^ High-intensity interval endurance training; ^11^ Moderate-intensity continuous endurance training; ^12^ Data shown as log; ^13^ Means ± standard error; ^14^ Means (range); ^15^ Adjusted values; ^16^ hsCRP; ^17^ Bonferroni correction; ^18^ Median (range); ^a^ Studies marked with the same letters were conducted in the same population.

**Figure 2:**
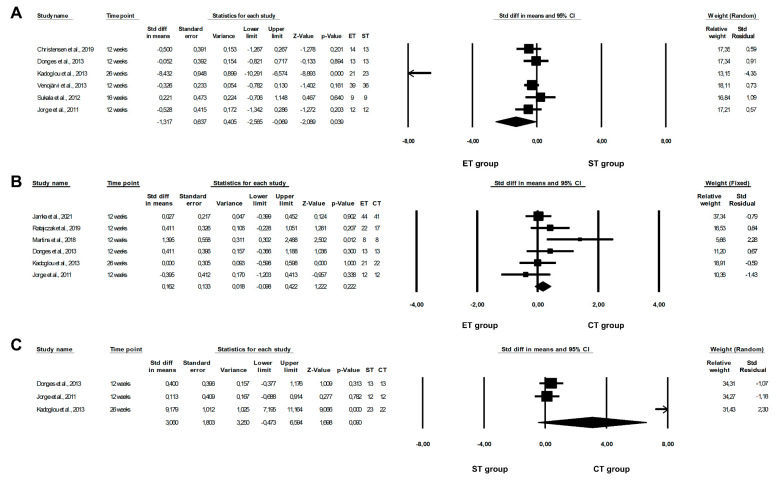
Forest plots of the effect of different training programmes on CRP levels: (**A**) ET vs. ST—random model; (**B**) ET vs. CT—fixed model; (**C**) ST vs. CT—random model. CI—confidence interval; CT—combined training; ET—endurance training; ST—strength training; Std diff—standard differences [14,15,16,21,22,23,25,40,48].

Upon conducting the meta-analysis, we found that an ET programme was significantly more beneficial in reducing CRP levels than an ST programme (ET vs. ST: random-effects model, SMD: −1.317, 95% CI: −2.565, −0.070, *p* = 0.0385, Figure 2A). However, the risk of heterogeneity among the included studies was high (Q-value = 74.169, *p* < 0.0001, I^2^ = 93.259%). There were no significant differences between ET and CT, or between ST and CT programmes, with regards to CRP concentrations (ET vs. CT: fixed-effects model, SMD: 0.106, 95% CI: −0.098, 0.422, *p* = 0.2215, Figure 2B; ST vs. CT: random-effects model, SMD: 3.060, 95% CI: −0.473, 6.594, *p* = 0.0896, Figure 2C). We observed no significant or high risk of heterogeneity among the included studies (ET vs. CT: Q-value = 8.3633, *p* = 0.1373, I^2^ = 40.215%; ST vs. CT: Q-value = 72.169, *p* < 0.0001, I^2^ = 97.229%). Moreover, when the fixed-effects model was used, significant differences between the effect of ST and CT programmes on CRP concentrations were also seen (SMD: 0.913, 95% CI: 0.377, 1.450, *p* = 0.0009, data not shown). 

### 3.6. Effect of Physical Training on IL-6 Concentrations 

In twelve publications, the effect of physical training on IL-6 levels was assessed [16,17,19,21,23,24,25,40,43,46,47,48]. Five of the studies compared the effect of ET and ST [17,19,21,24,40], four measured the effect of ET and CT [23,46,47,48], and three assessed the effect of ET, ST, and CT [16,25,43]. The changes between pre- and post-intervention values between endurance and combined groups were statistically significant only in one study [47].

**Figure 3:**
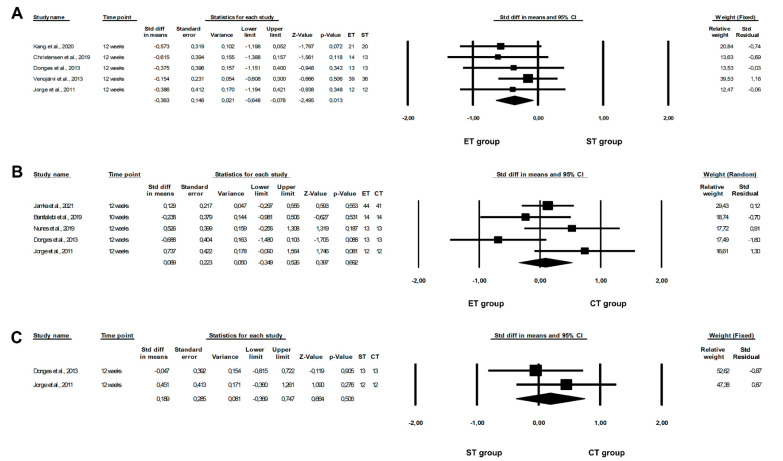
Forest plots of the effect of different training programmes on IL-6 levels: (**A**) ET vs. ST—fixed model; (**B**) ET vs. CT—random model; (**C**) ST vs. CT—fixed model. CI—confidence interval; CT—combined training; ET—endurance training; ST—strength training; Std diff—standard differences [16,21,23,24,25,40,46,47].

The meta-analysis revealed that an ET programme was more beneficial in lowering IL-6 concentrations than an ST programme (ET vs. ST: fixed-effects model, SMD: −0.363, 95% CI: −0.648, −0.078, *p* = 0.0126, Figure 3A) with low heterogeneity among the studies included (Q-value = 1.662, *p* = 0.7976, I^2^ = 0.000%). However, our meta-analysis did not confirm significant differences between ET and CT or between ST and CT programmes on IL-6 levels (ET vs. CT: random-effects model, SMD: 0.089, 95% CI: −0.349, 0.526, *p* = 0.6916, Figure 3B; ST vs. CT: fixed-effects model, SMD: 0.189, 95% CI: −0.369, 0.747, *p* = 0.5065, Figure 3C). In addition, we observed a moderate or very low risk of heterogeneity among the included studies (ET vs. CT: Q-value = 8.042, *p* = 0.0900, I^2^ = 50.264%; ST vs. CT: Q-value = 0.7616, *p* = 0.3828, I^2^ = 0.000%).

### 3.7. Effect of Physical Training on TNF-α Concentrations

The effect of exercise programmes on TNF-α concentrations was evaluated in ten studies [16,17,19,21,23,25,38,39,40,43]. Six papers assessed the effect of ET and ST [17,19,21,38,40,43], one study compared the effect of ET and CT [23], and three studies measured the effect of ET, ST and CT [16,25,39]. No differences between other training programmes were noted. 

**Figure 4:**
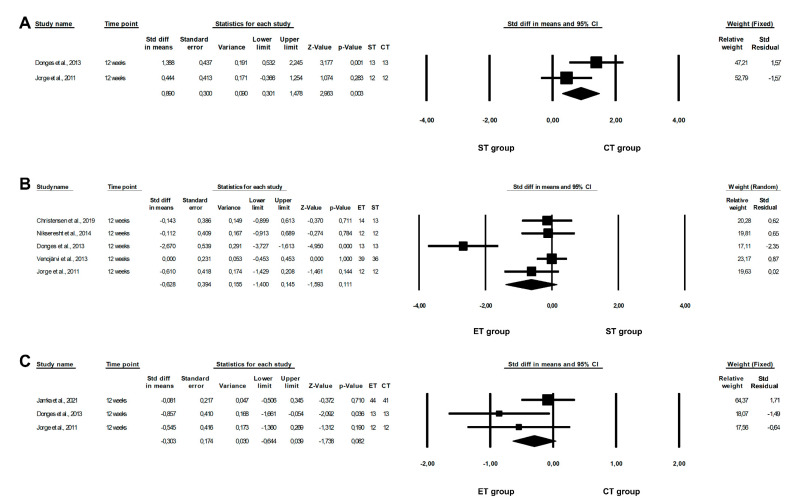
Forest plots of the effect of different training programmes on TNF-α levels: (**A**) ST vs. CT—fixed model; (**B**) ET vs. ST—random model; (**C**) ET vs. CT—fixed model. CI—confidence interval; CT—combined training; ET—endurance training; ST—strength training; Std diff—standard differences [16,21,23,25,38,40].

The results of this meta-analysis showed that a CT programme is significantly more beneficial in reducing TNF-α levels than an ST programme (ST vs. CT: fixed-effects model, SMD: 0.890, 95% CI: −0.301, 1.478, *p* = 0.0030, Figure 4A), and revealed a non-significant risk of heterogeneity among the studies included (ST vs. CT: Q-value = 2.467, *p* = 0.1162, I^2^ = 59.473%). However, there were no differences between the effect of ET and ST, or between ET and CT programmes, with regards to TNF-α levels (ET vs. ST: random-effects model, SMD: −0.628, 95% CI: −1.400, 0.144, *p* = 0.1112, Figure 4B; ET vs. CT: fixed-effects model, SMD: −0.303, 95% CI: −0.644, 0.039, *p* = 0.0823, Figure 4C), and the findings indicated a high or non-significant risk of heterogeneity among the studies included (ET vs. ST: Q-value = 21.808, *p* = 0.0002, I^2^ = 81.658%; ET vs. CT: Q-value = 3.215, *p* = 0.2004, I^2^ = 37.796%). Furthermore, when the fixed-effects model was calculated, significant differences were also observed between the effects of ET and ST programmes on TNF-α levels (SMD: −0.352, 95% CI: −0.660, 0.005, *p* = 0.0025). 

Funnel plots of standard error by standard differences in means of inflammatory parameters are presented in the Supplementary Materials (see Figures S1–S3).

### 3.8. Effect of Physical Training on Leptin Levels 

The effect of different training programmes on levels of adipokines is reported in Table 4. Four studies evaluated the effect of exercise on leptin concentrations [19,40,45,47]. Two studies compared the effect of ET and CT training, and two evaluated the effect of ET and ST [19,40]. One study found significant differences between ET and ST [45].

**Figure 5:**
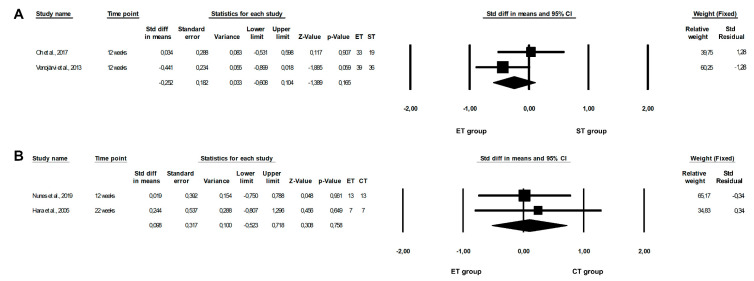
Forest plots of the effect of different training programmes on leptin levels: (**A**) ET vs. ST—fixed model; (**B**) ET vs. CT—fixed model. CI—confidence interval; CT—combined training; ET—endurance training; ST—strength training; Std diff—standard differences [19,40,45,47].

However, the results of the meta-analysis did not show any significant differences between the effect of ET and ST or between ET and CT programmes on leptin levels (ET vs. ST: fixed-effects model, SMD: −0.252, 95% CI: −0.608, 0.104, *p* = 0.1647, Figure 5A; ET vs. CT: fixed-effects model, SMD: 0.098, 95% CI: −0.523, 0.718, *p* = 0.7581, Figure 5B), and indicated no significant heterogeneity among the studies included (ET vs. ST: Q-value = 1.636, *p* = 0.2009, I^2^ = 38.879%; ET vs. CT: Q-value = 0.115, *p* = 0.7344, I^2^ = 0.000%).

### 3.9. Effect of Physical Training on Adiponectin Levels 

Ten studies compared the effect of different training programmes on adiponectin concentrations [19,20,21,22,25,40,41,42,44,45,47]. Five papers reported the effect of ET and ST [19,21,22,40,44], two studies compared the effect of ST and CT [45,47], and three studies examined the effects of ET, ST, and CT [20,25,41,42]. Two studies found significant differences between the effect of ET and concurrent training [45,47], and one study reported differences between ET and ST and between ST and CT [20].

**Figure 6:**
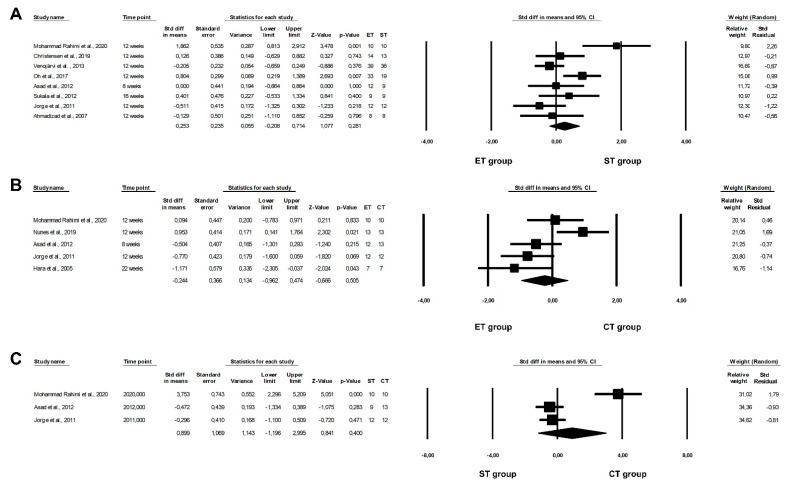
Forest plots of the effect of different training programmes on adiponectin levels: (**A**) ET vs. ST—random model; (**B**) ET vs. CT—random model; (**C**) ST vs. CT—random model. CI—confidence interval; CT—combined training; ET—endurance training; ST—strength training; Std diff—standard differences [19,20,21,22,25,40,41,42,45,47].

Nevertheless, this meta-analysis did not report any significant differences between the effects of ET and ST, ET and CT, or ST and CT programmes with regards to adiponectin concentrations (ET vs. ST: random-effects model, SMD: 0.235, 95% CI: −0.208, 0.714, *p* = 0.281, Figure 6A; ET vs. CT: random-effects model, SMD: −0.244, 95% CI: −0.962, 0.474, *p* = 0.5053, Figure 6B; ST vs. CT: random-effects model, SMD: 0.899, 95% CI: −1.196, 2.995, *p* = 0.4003, Figure 6C). In addition, we noted a moderate or high risk of heterogeneity among the studies included (ET vs. ST: Q-value = 20.565, *p* = 0.0045, I^2^ = 65.961%; ET vs. CT: Q-value = 13.377, *p* = 0.0096, I^2^ = 70.098%; ST vs. CT: Q-value = 26.661, *p* < 0.0001, I^2^ = 92.498%).

### 3.10. Effect of Physical Training on Visfatin Levels 

Four studies evaluated the effect of exercise programmes on visfatin levels [15,18,25,37]. All studies evaluated the effect of ET, ST, and CT. Two studies reported significant differences between the effect of ST and CT [15,37], one paper found significant differences between ET and CT training [18], and one between ET and ST [15]. 

**Figure 7:**
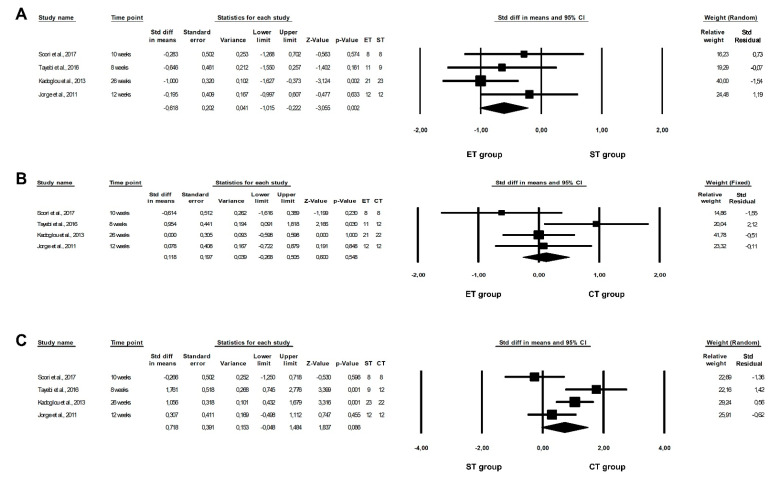
Forest plots of the effect of different training programmes on visfatin levels: (**A**) ET vs. ST—random model; (**B**) ET vs. CT—fixed model; (**C**) ST vs. CT—random model. CI—confidence interval; CT—combined training; ET—endurance training; ST—strength training; Std diff—standard differences [15,18,25,37].

ET programmes appeared to be significantly more beneficial in lowering visfatin concentrations than ST programmes (ET vs. ST: fixed-effects model, SMD: −0.618, 95% CI: −1.015, −0.222, *p* = 0.0023, Figure 7A). In addition, we observed a very low risk of heterogeneity among the studies included (Q-value = 2.940, *p* = 0.4010, I^2^ = 0.000%). However, our meta-analysis showed no differences between the effect of ET and CT or between ST and CT programmes on visfatin levels (ET vs. CT: fixed-effects model, SMD: 0.118, 95% CI: −0.268, 0.505, *p* = 0.5048, Figure 7B; ST vs. CT: random-effects model, SMD: 0.718, 95% CI: −0.048, 1.484, *p* = 0.0662, Figure 7C) and indicated a non-significant or moderate risk of heterogeneity among the studies included (ET vs. CT: Q-value = 5.808, *p* = 0.1213, I^2^ = 48.350%; ST vs. CT: Q-value = 9.987, *p* = 0.0187, I^2^ = 69.959%). Moreover, when the fixed-effects model was calculated, significant differences between the effect of ST and CT programmes on visfatin concentrations were also observed (SMD: −0.720, 95% CI: −0.351, 1.160, *p* = 0.0003).

**Table 4:** Adipokines levels in studied populations.

Author	Year	Group	Leptin [µg/mL]			Adiponectin [µg/mL]			Visfatin [ng/mL]		
			Pre	Post	Changes	Pre	Post	Changes	Pre	Post	Changes
Mohammad Rahimi et al. [20]	2020	ET	NI	NI	NI	2660 ± 820 ^2,3,4^	6520 ± 990 ^2,3,4^	3860 ^2^ (145.1% ^5^)	NI	NI	NI
		ST				2790 ± 940 ^2,3,4^	4420 ± 1250 ^2,3,4^	1670 ^2^ (59.85% ^5^)			
		CT				2700 ± 690 ^2,3,4^	6440 ± 680 ^2,3,4^	3700 ^2^ (137.04% ^5^)			
		CG				2660 ± 820 ^2,3,4^	2700 ± 860 ^2,3,4^	NI			
		*p * ^1^				ET, ST, CT: *p* <0.05 (pre vs. post) ET, ST, CT vs. CG, CT vs. ST, ET vs. ST: *p* < 0.05 (time × group interaction, post-hoc)					
Christensen et al. [21]	2019	ET	NI	NI	NI	NI	17.30 (14.94–19.65) ^2,6^	−0.69 (−3.04–1.67) ^2,6^	NI	NI	NI
								−2.5 ^7^ (−17.112.2)% ^5,6^			
		ST					16.79 (14.40–19.21) ^2,6^	−1.20 (−3.62–1.23) ^2,6^			
								−9.5 ^7^ (−24.7–5.7)% ^5,6^			
		CT					17.50 (14.81–20.18) ^2,6^	−0.49 (−3.17–2.20) ^2,6^			
								1.0 ^7^ (−15.8–17.8)% ^5,6^			
Nunes et al. [47]	2019	ET	22.57 (16.13–29.02) ^2,8^	17.59 (11.16–24.02) ^2,8^	−4.98 (−9.78–(−0.19)) ^2,8^	4.35 (3.02–5.68) ^2,8^	4.71 (3.59–5.82) ^2,8^	0.35 (−0.96–1.67) ^2,8^	NI	NI	NI
					−28.38 (−55.59–(−1.06))% ^2,5,8^			8.12 (−7.84–13.58)% ^2,5,8^			
		CT	21.33 (15.17–27.49) ^2,8^	17.42 (12.91–21.92) ^2,8^	−3.91 (−11.62–3.80) ^2,8^	5.12 (4.02–6.23) ^2,8^	3.00 (1.96–4.05) ^2,8^	−2.12 (−3.59–(−0.65))^2,8^			
					−18.34 (−54.49–17.8)% ^2,5,8^			−41.37 (−70.05–(−12.69))% ^2,5,8^			
		*p * ^1^	*p* = 0.043 (time)			*p* = 0.012 (time × group)					
Oh et al. [19]	2017	ET ^9^	NI	NI	−2.18 ± 0.89 ^4,11^ −3.2% ^5^	NI	NI	−0.016 ± 0.01 ^4,12^	NI	NI	NI
		ET ^10^			−1.45 ± 1.01 ^4,11^			−0.011 ± 0.018 ^4,12^			
		ST			−1.92 ± 0.68 ^4,11^ −14.3% ^5^			−0.026 ± 0.021 ^4,12^			
		*p * ^1^	ET ^9^, ST: *p* < 0.05 (pre vs. post)								
Soori et al. [18]	2017	ET	NI	NI	NI	NI	NI	NI	2.2 ± 0.8 ^4,11^	1.6 ± 0.6 ^4,11^	−0.62 ^2^ −28.3% ^5^
		ST							1.7 ± 1 ^4,11^	1.8 ± 0.8^4,11^	NI
		CT							2.8 ± 0.9 ^4,11^	2 ± 0.7 ^4,11^	−0.86 ^2^ −30.7% ^5^
		CG							2 ± 0.9 ^4,11^	1.7 ± 0.3 ^4,11^	NI
		*p * ^1^							*p* = 0.04 (post) CT: *p* = 0.003 (pre vs. post) ET: *p* = 0.045 (pre vs. post) CT vs. ET: *p* < 0.002 (post) CT vs. CG: *p* < 0.018 (post)		
Tayebi et al. [37]	2016	ET	NI	NI	NI	NI	NI	NI	NI	9.8 ± 0.4 ^4,13^	NI
		ST								10.7 ± 0.5 ^4,13^	
		CT								8.7 ± 0.3 ^4,13^	
		*p * ^1^							*p* = 0.005 (post) ST vs. CT: *p* = 0.004 (changes, post-hoc)		
Kadoglou et al. [15]	2013	ET	NI	NI	NI	NI	NI	NI	35 ± 8 ^4,11^	24 ± 9 ^4,11^	NI
		ST							31 ± 8 ^4,11^	33 ± 9 ^4,11^	
		CT							36 ± 8 ^4,11^	24 ± 8 ^4,11^	
		CG							31 ± 8 ^4,11^	30 ± 9 ^4,11^	
		*p * ^1^							*p* < 0.05 (changes, post-hoc) ET, CT vs. CG: *p* < 0.05, ET, CT vs. ST: *p* < 0.05		
Venojärvi et al. [40]	2013	ET	14.1 ± 2.8 ^14^	NI	−3.8 ± 1.2 ^14^	11.2 ± 1.3 ^14^	NI	0.0 ± 0.8 ^14^	NI	NI	NI
		ST	11.5 ± 1.4 ^14^		−0.9 ± 0.9 ^14^	10.4 ± 1.1 ^14^		0.9 ± 0.6 ^14^			
		CG	7.6 ± 1.3 ^14^		−0.2 ± 0.9 ^14^	12.1 ± 1.5 ^14^		0.2 ± 0.7 ^14^			
		*p * ^1^	*p* = 0.009 (pre) *p* = 0.001 (changes) ET vs. CG: *p* = 0.015 ^7^, ST vs. CG: *p* = 0.036 ^7^ (pre, post-hoc) ET vs. CG: *p* = 0.001 ^7^ (changes, post-hoc)								
Asad et al. [41,42]	2012	ET	NI	NI	NI	16.67 ± 2.35 ^11^	17.56 ± 1.51 ^11^	NI	NI	NI	NI
		ST				16.67 ± 2.35 ^11^	17.56 ± 1.51 ^11^				
		CT				17.00 ± 5.37 ^11^	20.38 ± 7.61 ^11^				
		CG				20.30 ± 8.35 ^11^	18.80 ± 2.69 ^11^				
Sukala et al. [22]	2012	ET	NI	NI	NI	6.7 ± 3.3 ^11^	6.7 ± 3.2 ^11^	0.1 ± 2.2 ^11^	NI	NI	NI
		ST				5.6 ± 1.9 ^11^	5.6 ± 2.2 ^11^	0.0 ± 1.4 ^11^			
Jorge et al. [25]	2011	ET	NI	NI	NI	5.58 ± 5.73 ^11^	3.38 ± 2.22 ^11^	NI	112.24 ± 45.83 ^11^	131.54 ± 58.38 ^11^	NI
		ST				4.45 ± 4.12 ^11^	5.13 ± 4.30 ^11^		112.11 ± 42.85 ^11^	142.25 ± 51.04 ^11^	
		CT				5.98 ± 3.43 ^11^	6.58 ± 5.44 ^11^		116.19 ± 75.41 ^11^	127.46 ± 45.22 ^11^	
		CG				5.07 ± 5.50 ^11^	3.75 ± 2.93 ^11^		103.57 ± 55.06 ^11^	134.12 ± 72.06 ^11^	
		*p * ^1^							ET, ST, CT, CG: *p* < 0.05 ^15^ (pre vs. post)		
Ahmadizad et al. [44]	2007	ET	NI	NI	NI	9.5 ± 3.4 ^4,11^	9.45 ± 1.1 ^4,11^	NI	NI	NI	NI
		ST				11.3 ± 1.4 ^4,11^	9.7 ± 2.5 ^4,11^				
		CG				10.3 ± 1.9 ^4,11^	12.1 ± 4.4 ^4,11^				
Hara et al. [45]	2005	ET	7.3 ± 2.8 ^11^	6.0 ± 2.6 ^11^	NI	3.7 ± 2.2 ^11^	4.0 ± 1.9 ^11^	NI	NI	NI	NI
		CT	5.9 ± 2.0 ^11^	5.4 ± 2.3 ^11^		6.2 ± 2.0 ^11^	6.6 ± 2.5 ^11^				
		CG	8.8 ± 2.3 ^11^	8.6 ± 2.5 ^11^		4.0 ± 1.2 ^11^	4.2 ± 1.3 ^11^				
		*p * ^1^	ET: *p* < 0.05 (pre vs. post) ET vs. ST, CG: *p* < 0.05 (pre, post-hoc)			CT vs. CG, ET: *p* < 0.05 (pre, post-hoc)					

CG—control group; CT—combined training; ET—endurance training; N/A—not applicable; NI—no information; post—after intervention; pre—before intervention; ST—strength training. ^1^ Only statistically significant values are shown; ^2^ Converted values; ^3^ High molecular weight-adiponectin; ^4^ Data from figure; ^5^ Relative changes; ^6^ Least square means (means adjusted for baseline) with (95% confidence intervals); ^7^ Value after Bonferroni; ^8^ correction Mean and 95% confidence intervals; ^9^ High-intensity interval endurance training; ^10^ Moderate-intensity continuous endurance training; ^11^ Mean ± standard deviation; ^12^ Data shown as log; ^13^ Adjusted mean ± standard error; ^14^ Mean ± standard error;^15^ ANOVA split-plot in time design.

Funnel plots of standard error by standard differences in means of adipokine levels are included in the Supplementary Materials (see Figures S4 and S5).

### 3.11. Risk of Bias

**Figure 8:**
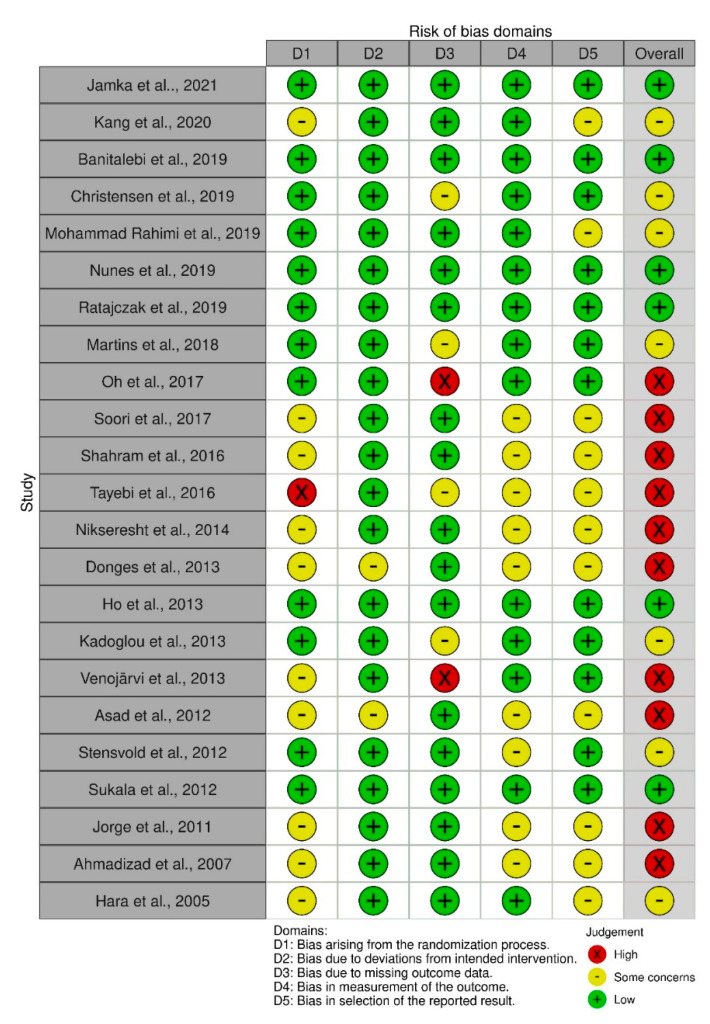
Traffic−light plot of the risk of bias [14,15,16,17,18,19,20,21,22,23,24,25,37,38,39,40,41,42,43,44,45,46,47,48].

**Figure 9:**
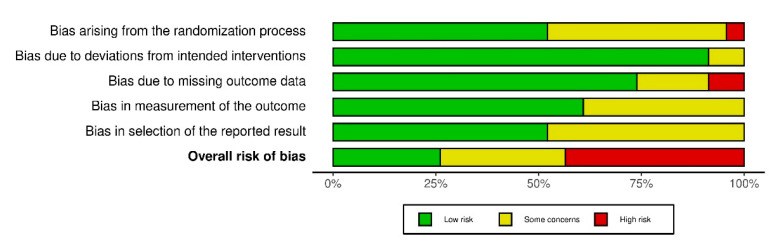
Summary plot of the risk of bias.

The results of the assessment for risk of bias are presented in Figure 8 and Figure 9. A high risk of bias was detected for nine studies [16,17,18,19,25,37,38,40,41,42]. There were some concerns about bias in seven studies [15,20,21,24,43,45,48], while six studies had a low risk of bias [14,22,23,39,46,47]. Twelve studies described the randomisation process and allocation concealment in sufficient detail to be rated as low risk of bias arising from the randomisation process [14,15,19,20,21,22,23,39,43,46,47,48]. Although the blinding of participants and study personnel was not possible in most of the studies, due to the nature of the intervention, twenty-one studies were judged to be at low risk of bias due to deviations from intended intervention [14,15,17,18,19,20,21,22,23,24,25,37,38,39,40,43,44,45,46,47,48]. Seventeen studies were rated as low risk of bias due to missing outcome data [14,16,17,18,20,22,23,24,25,38,39,41,42,43,44,45,46,47]. Fourteen studies used appropriate methods for the outcomes measured and, therefore, were assessed as low risk of bias [14,15,19,20,21,22,23,24,39,40,45,46,47,48]. Finally, twelve studies had a low risk of bias in their selection of the reported results [14,15,19,21,22,23,39,40,43,46,47,48].

## 4. Discussion

Our study incorporated 24 trials which included data from 1145 overweight and obese adults. The effects of ET, ST, and CT were compared by assessing their influence on the levels of inflammatory markers (CRP, IL-6, and TNF-α) and adipokines (leptin, adiponectin, and visfatin). The results of our meta-analysis clearly show a more beneficial effect of ET training in reducing CRP, IL-6, and visfatin levels, compared with ST. Moreover, our study indicates that CT is more effective in reducing TNF-α levels compared with ST alone. However, we did not identify any differences between the effects of different training programmes on adiponectin and leptin concentrations.

Previously, it has been shown that lifestyle interventions aiming to reduce weight in overweight or obese adult populations reduce mortality, regardless of their success in achieving weight loss [49]. It is now also believed that adipose tissue is an active endocrine organ that secretes various adipokines and pro-inflammatory cytokines, which, in obesity, can lead to a low level of systemic inflammation [50]. It is also associated with changes in levels of CRP, which is produced mainly by the liver in a response to pro-inflammatory cytokines, such as IL-6 and TNF-α, but has also been shown to be produced in adipose tissue and atherosclerotic plaques [51]. A recent meta-analysis comparing the independent effects of ET, ST, and CT on subcutaneous abdominal adipose tissue (SAT) in adults has shown that all these types of training lead to SAT reduction, while endurance exercise was shown to produce the greatest efficacy in decreasing SAT [52]. Several systematic reviews and meta-analyses have provided evidence of improvement in some inflammatory markers after different training sessions in various populations [26,53,54,55,56,57]. 

Focusing on meta-analyses, Zheng et al. [26] have shown that ET significantly decreased CRP, TNF-α, and IL-6 without reducing IL-4 levels in healthy middle-aged and elderly people when compared to the control group. Meanwhile, Hayashino et al. [53] assessed the effects of any type of supervised exercise (endurance, strength, and combined) or physical exercise advice on inflammatory markers and adipokine levels in adults with T2D, and observed that training, overall, resulted in improved IL-6 and CRP comparing to the inactive control group in this population. Moreover, this exercise was more effective in lowering IL-6 levels where programmes had longer durations and a greater number of sessions. A meta-analysis by Monteiro-Junior et al. [54] has also shown a significant reduction in IL-6 and CRP concentrations, but not TNF-α levels, after chronic overall exercise intervention in older adults. However, Meneses-Echávez et al. [55] conducted a meta-analysis evaluating the influence of overall exercise training on mediators of inflammation in breast cancer survivors, and only observed improvement in the concentrations of IL-6, TNF- α, IL-8, and IL-2, without any differences in the concentrations of CRP when compared to a control group who received no intervention. Finally, Khalafi et al. [56], in their meta-analysis, compared the effect of exercise alone versus caloric restriction alone, as well as exercise combined with caloric restriction versus caloric restriction alone, on inflammatory parameters in overweight and obese subjects, and showed that a combination of exercise with caloric restriction may be more effective than caloric restriction alone, causing a larger decrease in IL-6 and TNF-α, and tending to decrease CRP in this population. The most recent meta-analysis by Khalafi et al. [57] indicated that not only overall exercise but also ET, ST, and CT alone significantly reduced IL-6, TNF-α, and CRP concentrations in postmenopausal women when compared to an inactive control group. On the other hand, other systematic reviews and meta-analyses have not indicated an effective reduction in IL-6 and TNF-α concentrations after chronic overall exercise [54] or resistance training [58] in older adults compared to a control group who received no exercise intervention. Previous studies only assessed the overall effect of exercise, and the authors did not focus on comparing the effects of different types of training on the levels of inflammatory parameters. Only some of the works presented additional subgroup analysis to check whether a given type of training influences the inflammatory parameters, but even these did not compare the individual types of training. In our meta-analysis, we focused on the comparison of ET, ST, and CT, and indicated that ET has a more beneficial effect in reducing CRP and IL-6 levels in this population compared with ST alone. Moreover, we observed that CT is more beneficial than ST in reducing TNF-α levels.

The differences between the effects of particular types of training on inflammatory parameters may be explained by the promotion of other specific cardiovascular and neuromuscular adaptations [59]. ET causes adaptations of the musculoskeletal and cardiovascular systems that support an increase in performance and exercise capacity [60], while ST promotes neuromuscular adaptations that lead to power development and muscle strength improvement [61]. On the other hand, CT, as a combination of ET and ST, is a promising way to increase performance by training both cardiorespiratory fitness and muscle strength [62]. In general, our findings align with other studies, indicating positive effects of exercise on inflammatory parameters, and reinforcing the appropriateness of exercise prescription for different populations. Moreover, they indicate that, in order to obtain better therapy effects, it is important to select the appropriate type of training. 

Being overweight or obese contributes to increased leptin and visfatin levels and decreased adiponectin concentrations [63,64]. A lot of studies have shown the positive effect of exercise on levels of adipokines in adults [53,57,65,66,67], and in the paediatric population [68,69]. However, the evidence from these studies has not been conclusive. In a systematic review and meta-analysis investigating possible beneficial effects of exercise on adiponectin and leptin levels in overweight and obese subjects, Yu et al. [65] revealed that exercise, particularly endurance training, significantly increased adiponectin and reduced serum leptin concentrations compared to a control. Another meta-analysis assessing the influence of exercise on adipokine levels in adults with T2D has shown that overall exercise did not alter adiponectin or leptin concentrations, and that only an ET program was associated with a significant change in leptin levels [53]. However, overall exercise has been shown to be effective in increasing adiponectin compared to a control in postmenopausal women [57], while, in a meta-analysis of the adult population, adipokine levels also increased after ST [66]. Moreover, in the meta-analysis by Rostás et al. [67], ST appeared to be more efficient in reducing leptin concentrations than ET alone in middle-aged or older overweight or obese subjects. On the other hand, in a meta-analysis assessing the influence of exercise on adipokine levels in the obese paediatric population, CT resulted in greater increases in adiponectin levels than ET alone [68], while leptin concentrations decreased significantly after both ET and CT [69]. Previous studies show that different types of training can positively affect leptin and adiponectin levels, but there are no clear conclusions as to which type of training most effectively improves the concentrations of these adipokines. In our meta-analysis, we did not find any differences between the effect of ET, ST, and CT on leptin and adiponectin concentrations in overweight and obese adults. A possible explanation for our results is that the changes in these parameters are not related to the type of exercise, but to the change in body weight [70,71] or the duration [72,73] and intensity [74] of the intervention. In a three-year weight loss study, Madsen et al. [70] indicated that weight loss greater than 10% can improve the levels of circulating adiponectin, as well as inflammatory parameters in obese subjects. A greater than 10% increase in adiponectin levels after weight loss was also confirmed in older obese adults with and without periodontal disease, 3–18 months post enrolment [71]. It is accepted that chronic exercise resulting in weight reduction corresponds to an increase in adiponectin concentrations [75] and a decline in leptin levels [76]. Studies on the effects of acute exercise and the corresponding changes or lack thereof in leptin levels are less conclusive. In the study by Weltman et al. [72], 30 min of exercise at various intensities and caloric expenditures did not appear to be sufficient to affect leptin concentrations during exercise, or 3.5 h after training in healthy young men. On the other hand, Nindl et al. [73] indicated that, after acute strength exercises with an energy expenditure of 855 ± 114 kcals, leptin concentrations were lower compared to the control nine hours following the exercise. Moreover, in an RTC on overweight inactive elderly subjects, Fatouros et al. [74] indicated that, after six months of ST followed by six months of detraining, leptin concentrations decreased after low, moderate, and intensive ST, whereas adiponectin levels increased only after intensive ST. Furthermore, the small number of studies included in our analysis may also reinforce the need for more trials to confirm which type of training has a better effect on adipokines. 

Our results do indicate that ET was more effective at lowering visfatin levels than ST. This may be related to the fact that ET leads to greater adipocyte tissue loss than ST, which in turn leads to a greater decrease in visfatin concentrations [52]. There is a lack of meta-analysis assessing the influence of exercise on visfatin concentrations in the adult population; however, a review of paediatric obesity indicated that overall exercise has an impact on the release of visfatin in this population [77]. Some intervention studies have evaluated the influence of different training on visfatin levels. Twelve-week CT intervention has been found to be effective in reducing visfatin levels in middle-aged obese women [78], while the same duration of ET did not significantly change visfatin levels in these obese women [79]. On the contrary, another researcher reported that twelve weeks of ET reduced the levels of visfatin in obese young subjects, with T2D or normal glucose tolerance [80]. Our results assessing the effect of different training programmes on visfatin levels should be interpreted with caution, due to the small number of studies included in the analysis.

To our knowledge, this is one of the first meta-analyses comparing the effect of ET, ST, and CT on inflammatory markers and levels of adipokines in overweight and obese adults. The other strengths of this meta-analysis include the detailed characteristics of the studies included and study populations, as well as specific inclusion and exclusion criteria. In addition, excluding studies with any dietary consultation or intervention allowed us to evaluate the actual impact of various training programmes on inflammatory markers and adipokine levels in overweight and obese adults. Moreover, this meta-analysis was written based on a search of PubMed, Web of Science, Scopus, and Cochrane, which are the largest and the most available databases. Furthermore, double counting of subjects from overlapping publications was prevented during the meta-analysis. However, heterogeneity was still significant owing to differences in the lengths, types, and durations of the exercise interventions, and the participants involved in the studies. Significant heterogeneity may also be related to different sampling and preparation methods, as well as the time elapsed between previous exercise sessions and sample measurements, which may affect inflammatory markers and adipokine levels. Another limitation of our publication is that the availability of outcome data that could be used for meta-analysis was limited, and the information needed was not always provided by the authors after contact. Moreover, for comparisons of ST versus CT, there was a lack of studies showing leptin concentrations suitable for meta-analysis. Other limitations include the fact that only six studies had a low risk of bias, while seven studies were assessed as having some concerns about bias, and a high risk of bias was found in nine studies. This could have impacted our analysis of the actual effects of the interventions. However, due to the specificity of the exercise interventions, it was not possible to conduct double-blind trials; therefore, a performance bias may be unavoidable in studies of this nature. In addition, several studies evaluated in the meta-analysis included subjects with comorbidities, such as DM2, prediabetes, or metabolic syndrome. However, these are common diseases in overweight and obese people. To reduce heterogeneity, we disqualified studies involving subjects with rare comorbidities unrelated to obesity, such as cancers, lung, musculoskeletal, or gastrointestinal diseases. Another limitation of our study is the lack of subgroup analysis in terms of the duration of the intervention, body weight, or training intensity, which was not possible due to the small number of studies included in the meta-analysis. Moreover, we did not perform a sensitivity or meta-regression analysis to remove the sources of heterogeneity or variance in the studies included in the meta-analysis.

Our meta-analysis indicates the effectiveness of exercise therapy for reducing inflammatory markers and adipokine levels in overweight and obese adults. However, considering the limited number of included studies and the fact that we did not identify any differences between the effects of particular training programmes on adiponectin and leptin concentrations, we see a need for randomised control trials with larger sample sizes to determine the most suitable method to reduce levels of these adipokines. Moreover, the potential anti-inflammatory effects of the compared training programmes should be considered in future meta-analyses to clarify the influence of individual training programmes on inflammatory markers in subjects of different ages and with specific diseases.

## 5. Conclusions

In summary, this systematic review and meta-analysis of randomised control trials provides evidence that an endurance training programme is more beneficial in reducing CRP, IL-6, and visfatin concentrations in overweight and obese adults compared with a strength training programme. Additionally, a combined training programme appeared to be significantly more beneficial in lowering TNF-α levels compared with a strength training programme. Therefore, in the obese and overweight adult population, our findings suggest that training programmes including only strength exercise are the least appropriate for reducing inflammatory parameters and adipokine levels. However, we found no difference between the effects of different types of training on adiponectin and leptin concentrations, which may be related to the small number of studies included in the meta-analysis. Further randomised control trials need to be conducted to determine which type of training has a greater effect on the levels of these adipokines in the obese or overweight adult population.

## Data Availability

Template data collection forms, data extracted from included studies, data used for analysis, analytic code, and any other materials used in the review are available on reasonable request from the corresponding author (J.W.).

## Supplementary Materials

The following supporting information can be downloaded at: https://www.mdpi.com/article/10.3390/healthcare10061098/s1, Figure S1: Funnel plot of standard error by standard differences in means of CRP concentrations: (A) ET vs. ST; (B) ET vs. CT; (C) ST vs. CT in selected randomised trials; Figure S2: Funnel plot of standard error by standard differences in means of IL-6 concentrations: (A) ET vs. ST; (B) ET vs. CT in selected randomised trials; Figure S3: Funnel plot of standard error by standard differences in means of TNF-α concentrations: (A) ET vs. ST; (B) ET vs. CT; (C) ST vs. CT in selected randomised trials; Figure S4: Funnel plot of standard error by standard differences in means of adiponectin concentrations: (A) ET vs. ST; (B) ET vs. CT; (C) ST vs. CT in selected randomised trials; Figure S5: Funnel plot of standard error by standard differences in means of visfatin concentrations: (A) ET vs. ST; (B) ET vs. CT; (C) ST vs. CT in selected randomised trials.
